# Effects of Anesthesia Techniques on Outcomes after Hip Fracture Surgery in Elderly Patients: A Prospective, Randomized, Controlled Trial

**DOI:** 10.3390/jcm9061605

**Published:** 2020-05-26

**Authors:** Seokyung Shin, Seung Hyun Kim, Kwan Kyu Park, Seon Ju Kim, Jae Chan Bae, Yong Seon Choi

**Affiliations:** 1 Department of Anesthesiology and Pain Medicine, Anesthesia and Pain Research Institute, Yonsei University College of Medicine, Seoul 03722, Korea; skshin@yuhs.ac (S.S.); anesshkim@yuhs.ac (S.H.K.); 2 Department of Orthopedic Surgery, Yonsei University College of Medicine, Seoul 03722, Korea; kkpark@yuhs.ac; 3 Department of Anesthesiology and Pain Medicine, National Health Insurance Service Ilsan Hospital, Goyang 10444, Korea; sjkim603@nhimc.or.kr (S.J.K.); bjchn89@yuhs.ac (J.C.B.)

**Keywords:** hip fracture, elderly, anesthesia, HMGB1, IL-6, mortality

## Abstract

The superiority of distinct anesthesia methods for geriatric hip fracture surgery remains unclear. We evaluated high mobility group box-1 (HMGB1) and interleukin-6 (IL-6) with three different anesthesia methods in elderly patients undergoing hip fracture surgery. Routine blood test findings, postoperative morbidity, and mortality were assessed as secondary outcome. In total, 176 patients were randomized into desflurane (*n* = 60), propofol (*n* = 58), or spinal groups (*n* = 58) that received desflurane-based balanced anesthesia, propofol-based total intravenous anesthesia (TIVA), or spinal anesthesia, respectively. The spinal group required less intraoperative vasopressors (*p* < 0.001) and fluids (*p* = 0.006). No significant differences in HMGB1 (*p*_group×time_ = 0.863) or IL-6 (*p*_group×time_ = 0.575) levels were noted at baseline, postoperative day (POD) 1, or POD2. Hemoglobin, albumin, creatinine, total lymphocyte count, potassium, troponin T, and C-reactive protein were comparable among groups at all time-points. No significant differences in postoperative hospital stay, intensive care unit (ICU) stay, and ventilator use among groups were observed. Postoperative pulmonary, cardiac, and neurologic complications; and in-hospital, 30-day, and 90-day mortality were not significantly different among groups (*p* = 0.974). In conclusion, HMGB1 and IL-6, and all secondary outcomes, were not significantly different between desflurane anesthesia, propofol TIVA, and spinal anesthesia.

## 1. Introduction

Hip fracture is a surgical disease, with more than 95% of patients undergoing operative treatment [1]. The majority of hip fractures are complicated by the fact that the patient population is geriatric with various underlying medical conditions. In Europe and the USA, the 1-month mortality rate after hip fracture is reported to be between 4% and 12%, and as high as 35% after 1 year [2]. To improve patient outcomes, substantial research efforts are being enacted.

Two important facets of the surgical management of elderly hip fracture patients are the utility of laboratory tests for predicting outcomes and optimal anesthetic method. Several laboratory tests have been suggested to be predictive of mortality after hip fracture surgery, which include routine blood tests such as hemoglobin (Hb), total lymphocyte count (TLC), albumin, and creatinine [3]; and cytokines such as tumor necrosis factor-α (TNF-α), interleukin-6 (IL-6), and interleukin-10 (IL-10) [4]. Notably, the superiority of different methods of anesthesia in terms of clinical outcomes after hip fracture surgery is the focus of many researchers in the field. In this context, we focused on high mobility group box-1 (HMGB1), which acts as a damage-associated molecular pattern molecule that mediates the noninfectious inflammatory response, and its significance in traumatic hip fracture surgery. The clinical significance of HMGB1, a relatively novel inflammatory cytokine, has been reported in various diseases including cancer, inflammatory diseases, autoimmune diseases, and trauma [5]. While studies have reported greater benefit with regional anesthesia over general anesthesia, recent data suggest that clinical outcomes between these anesthetic methods are not significantly different [6].

The primary purpose of this study was to evaluate the potential differences in HMGB1 and IL-6 between elderly patients undergoing surgical treatment for hip fractures with either general or spinal anesthesia. To further differentiate between anesthesia methods, general anesthesia was divided into desflurane-based balanced anesthesia or total intravenous anesthesia (TIVA) with propofol. As secondary outcomes, we investigated whether different anesthesia methods would lead to a difference in morbidity and mortality after hip fracture surgery, and additionally analyzed routine laboratory test results that had been previously reported to be associated with outcome in hip fracture patients.

## 2. Materials and Methods

### 2.1. Study Population and Anesthesia Procedure

The study protocol was reviewed and approved by the Institutional Review Board and Hospital Research Ethics Committee of Severance Hospital, Yonsei University Health System (#4-2015-0088) and registered at http://clinicaltrials.gov (NCT02458547). Written informed consent was obtained from all patients. Patients over the age of 65 years that received daytime hip fracture surgery between May 2015 and January 2019 were screened and enrolled in this study. Patients with absolute contraindications to spinal anesthesia, known allergies to propofol, and altered mental status due to intracranial lesions were excluded. Patients previously diagnosed with dementia or those showing signs of delirium were enrolled after consent was obtained from a legal representative. Attempts to obtain consent from such patients were continuously made throughout the study period when the patient was deemed lucid per the guidelines of our Hospital Research Ethics Committee.

Patients were randomly assigned to either the desflurane, propofol, or spinal groups on the day of surgery according to a random table generated by a computer program. Patient allocation was performed by an investigator that was not involved in postoperative assessment. The investigator in charge of postoperative follow-up remained blinded to the method of anesthesia.

Upon arrival at the operating room, electrocardiography, non-invasive blood pressure, and pulse oximetry monitors were applied. Arterial cannulation for invasive blood pressure monitoring was performed when deemed necessary by the attending anesthesiologist. In the desflurane group, patients received pentothal sodium, cisatracurium, and remifentanil for induction. Anesthesia was maintained with desflurane in oxygen-air mixture (40:60) and remifentanil infusion. Patients in the propofol group received propofol, remifentanil, and cisatracurium for anesthesia induction and were maintained with target-controlled infusion (TCI) of propofol and remifentanil based on the Marsh [7] and Minto [8] model for the two drugs, respectively. In patients that underwent general anesthesia, intraoperative bispectral index (BIS) monitors were used. BIS levels were maintained between 40 and 60. Patients in the spinal group received spinal anesthesia with hyperbaric bupivacaine and were administered 3 L of O_2_ via nasal prong. The dose of hyperbaric bupivacaine used was 9 mg in patients shorter than 160 cm, and 11 mg in those 160 cm or taller. Patients that requested or required intraoperative sedation were administered midazolam starting at doses of 0.02 mg/kg.

A significant decrease in blood pressure (greater than 20% decrease in mean arterial pressure from baseline) was considered hypotension and was initially treated with a bolus of ephedrine (4 mg) up to two times. If hypotension persisted, additional ephedrine, phenylephrine, or norepinephrine was administered at the discretion of the attending anesthesiologist based on the patient’s vital signs and underlying medical conditions.

### 2.2. Outcome Assessment

HMGB1 and IL-6 were measured three times: on the morning of surgery as baseline, postoperative day (POD) 1 and POD2. Peripheral blood samples were centrifuged immediately after collection. Serum was frozen and stored at −80 °C until analysis. Serum HMGB1 was quantified by enzyme-linked immunosorbent assay (ELISA) using the HMGB1 ELISA kit (IBL International, Hamburg, Germany) according to the manufacturer’s instructions. Plasma IL-6 was analyzed using the Human IL-6 Quantikine ELISA Kit (R&D Systems, Inc., Minneapolis, MN).

Hb, TLC, creatinine, albumin, potassium, troponin T, and C-reactive protein (CRP) levels were compared among groups at three-time points: on the morning of surgery as baseline, POD1, and POD2. N-terminal pro-brain natriuretic peptide (NT-proBNP) level was evaluated once at baseline.

Intraoperative fluid input, blood loss, and amount of transfusion were recorded alongside the use of intraoperative vasopressors. Postoperative complications were categorized as pulmonary (pneumonia, respiratory failure), cardiac (myocardial infarct, heart failure, new-onset arrhythmia), and neurologic (aggravation or new onset of delirium). Postoperative hospital stay, duration of intensive care unit (ICU) admission, and ventilator use were also recorded and compared among groups. Mortality rates were assessed during the postoperative period at the hospital and up to 90 days after surgery.

### 2.3. Statistical Analysis

Sample size calculation was performed based on a previous study that quantified the concentration of HMGB1 in patients sustaining femur fractures after blunt trauma [9]. HMGB1 level on day 3 after admission was 53 ± 17 ng/mL in patients whose femoral fractures were stabilized by surgery within 24 h of injury. Under the assumption that preoperative baseline HMGB1 levels would be comparable between groups, we hypothesized that a 10 ng/mL difference would be clinically significant on POD2. To obtain 90% power with an α of 0.05, a sample size of 62 per group was required, allowing for a dropout rate of 10%.

Continuous variables that were normally distributed were analyzed with ANOVA, and multiple comparisons were performed with an independent t-test. Variables that were not normally distributed were analyzed with the Kruskal-Wallis test, and multiple comparisons were performed using the Dunn procedure. Repeated measures variables were analyzed using a linear mixed model with patient indicator as a random effect and group, time, and group-by-time as fixed effects. The group-by-time interaction assessed whether the change in variables over time differed between groups. Values are presented as mean (SD), median (interquartile range, IQR), mean (SEM), or number of patients (proportion) as appropriate. A *p*-value < 0.05 was considered statistically significant. All analyses were conducted using R package, version 3.4.4 (The R Foundation for Statistical Computing, Vienna, Austria), SAS version 9.4 (Cary, NC, USA), and SPSS Statistics version 25 (Armonk, NY, USA).

## 3. Results

### 3.1. Study Population, Demographic Data, and Perioperative Characteristics

**Figure 1:**
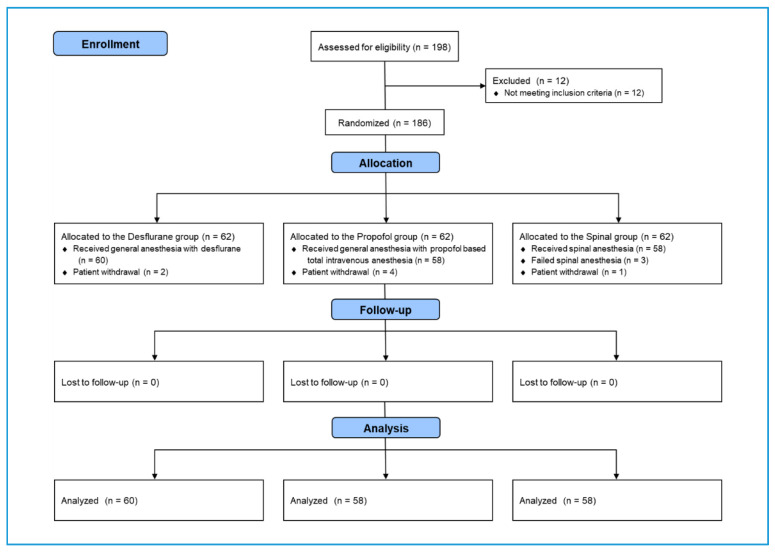
CONSORT (Consolidated Standards of Reporting Trials) flowchart of patient sample selection.

The CONSORT (Consolidated Standards of Reporting Trials) flow diagram is shown in Figure 1. A total of 198 patients admitted for surgery for femur fractures were assessed for eligibility. Twelve patients were excluded for not meeting the inclusion criteria. The 186 remaining patients were randomly allocated to the desflurane, propofol, and spinal groups. Two, four, and one patient in the desflurane, propofol, and spinal groups, respectively, withdrew from the study. An additional three patients were excluded from the spinal group when the spinal block failed, and the patient received a different type of anesthesia. None of the patients were lost to follow-up after surgery. The final analysis comprised 60, 58, and 58 patients in the desflurane, propofol, and spinal groups, respectively.

Patient characteristics are shown in Table 1. No differences were observed in patient age, sex, body mass index, or underlying comorbidities between the three groups. Baseline NT-proBNP, type of fracture, and time from injury to surgery were also similar between groups.

Operative characteristics are shown in Table 2. All patients underwent either bipolar hemiarthroplasty or internal fixation of the femur. No difference in type of surgery among the three groups was noted. Anesthesia time was comparable between groups. Patients in the spinal group required significantly less intraoperative vasopressors (*p* < 0.001) and were administered less intraoperative fluids (*p* = 0.006) than that of the other two groups. The remaining variables were otherwise unremarkable.

### 3.2. Proinflammatory Cytokines and Routine Laboratory Tests

**Figure 2:**
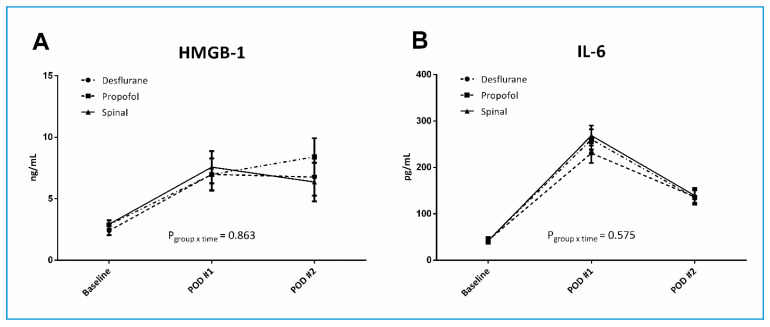
Proinflammatory cytokines in elderly patients undergoing hip fracture surgery. High mobility group box-1 (HMGB1) (**A**) and interleukin-6 (IL-6) (**B**) levels at baseline, postoperative day (POD)1, and POD2 of patients in the three groups. The whiskers represent the SEM.

Serum cytokine levels at three time-points are shown in Figure 2. HMGB1 levels were comparable between the three groups at all-time points. Changes in HMGB1 levels over time were similar in all groups (*p*_group×time_ = 0.863). The only difference observed was a decrease in HMGB1 level on POD2 in the desflurane and spinal groups, whereas it continued to increase in the Propofol group. IL-6 levels were similar among groups at all-time points. No changes over time were observed (*p*_group×time_ = 0.575). IL-6 levels peaked on POD1 and showed a decreasing trend on POD2.

**Figure 3:**
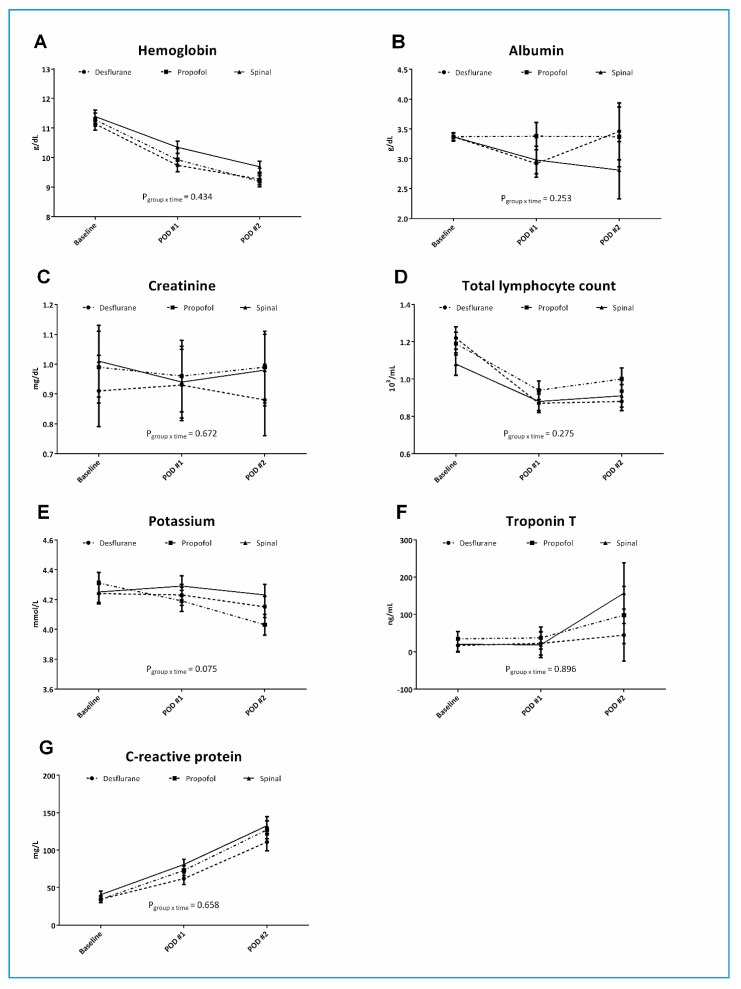
Routine laboratory test results in elderly patients undergoing hip fracture surgery. Hemoglobin (**A**), albumin (**B**), creatinine (**C**), total lymphocyte count (**D**), potassium (**E**), troponin T (**F**), and C-reactive protein (**G**) levels at baseline, postoperative day (POD)1, and POD2 of patients in the three groups. The whiskers represent the SEM.

Results of routine laboratory tests including Hb, albumin, creatinine, TLC, potassium, troponin T, and CRP are shown in Figure 3. No significant differences were observed in any of these variables among groups over time. A clear trend of decreasing Hb was observed in all three groups without significant differences between groups (*p*_group×time_ = 0.434). Variables that continued to increase from baseline to POD2 were troponin T and CRP.

### 3.3. Postoperative Morbidity and Mortality

**Figure 4:**
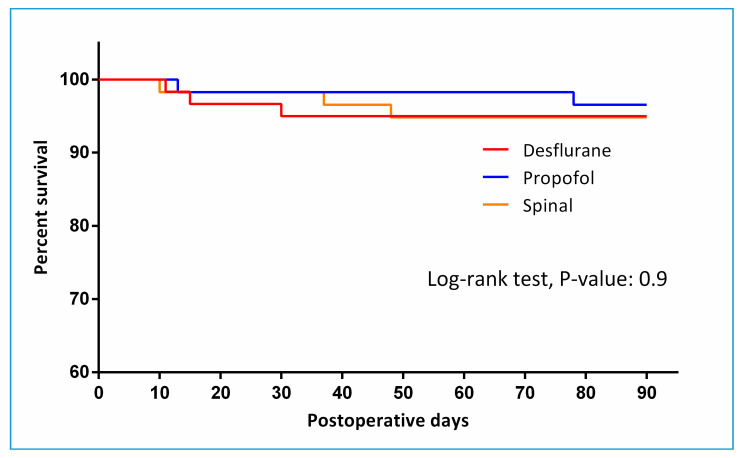
Kaplan–Meier curve showing survival up to 3 months postoperatively.

The duration of hospital stay was similar between the three groups, the median duration being approximately 1 week. The duration of ICU stay, ventilator use and postoperative stay were also all comparable between groups. The incidence of pulmonary, cardiac complications, and delirium; and in-hospital, 30-day, and 90-day, mortality were not significantly different among groups (Table 3). Figure 4 depicts 90-day survival (*p* = 0.974).

## 4. Discussion

Hip fracture surgery is the most common reason for urgent surgery in the geriatric population, and has been termed the “quintessential geriatric illness” [10]. In this vulnerable patient population, it is crucial to optimize each component of medical care. One such component is the method of anesthesia. The most recent systematic reviews to date conclude that clinical outcomes are not significantly different between modes of anesthesia, and the present study supports these previous findings [6,11,12]. We further categorized general anesthesia into volatile anesthetic-based balanced anesthesia (desflurane in this case), or TIVA with propofol, but did not observe any differences in HMGB1 or IL-6 between anesthesia methods. Other routine laboratory results that were assessed as secondary outcome were also not different between groups, as well as postoperative clinical outcomes.

For several decades, it was unclear whether general or regional anesthesia was favorable for hip fracture surgery outcomes, and this issue remains under debate. Currently, two large scale, multicenter randomized clinical trials (RCTs) comparing outcomes after spinal vs. general anesthesia for hip fracture surgery are underway [2,13], which will provide valuable data and insight regarding this issue. A limited number of RCTs to date have compared outcomes after different anesthesia techniques in hip fracture patients. Although it should be noted that anesthetics used in several studies differ substantially from those commonly used at present, two earlier studies of more than 500 patients both concluded that there were no differences in morbidity or mortality between spinal and general anesthesia for hip fracture surgery [14,15]. Two more recent RCTs [16,17] also reported the same conclusion, although both enrolled patients 50 years or older. Similar results were reported in major retrospective [18] and observational [19] studies, where 18,158 and 65,535 patients were analyzed, respectively. The novelty of our study is that it is of the first RCTs to compare not only general vs. spinal anesthesia, but also inhalational anesthesia with TIVA in elderly hip fracture patients.

The primary goal of the present study was to assess differences in the cytokines, HMGB1 and IL-6, among desflurane-based balanced anesthesia, propofol-based TIVA, and spinal anesthesia. The two cytokines were selected based on the extensive literature on IL-6 and more recent interest in HMGB1. The role of IL-6 in chronic inflammation and autoimmune diseases is well established. IL-6 has been proposed as a marker for predicting outcomes after hip fracture in elderly patients [4,20]. As a mediator of sterile inflammation and infection, HMGB1 has an important role in injury-elicited inflammatory diseases including trauma [9,21,22], but evidence in elderly hip fracture patients is lacking. The significance of HMGB1 in clinical practice has been reported in countless disease states, which include various forms of trauma [5]. HMGB1 has been suggested to have prognostic value in severe trauma patients [23], and also to be an indicator of the onset of multiple organ dysfunction syndrome in multiple trauma [24]. Moreover, surgical and anesthesia trauma has also been reported to elicit an increase in HMGB1, and the authors suggested a positive correlation between the release of IL-6 and HMGB1 following traumatic insult [25]. On the other hand, certain anesthetics including propofol [26], lidocaine [27,28], and remifentanil [29], were reported to inhibit the release of HMGB1. Based on these previous findings, we asked whether different anesthetic techniques would be able to lead to a difference in perioperative HMGB1 levels in elderly hip fracture patients undergoing surgical treatment. However, it was unclear how the preceding trauma of the fracture and the subsequent surgical/anesthesia would interact in terms of the release of HMGB1 and IL-6, and this is beyond the scope of our study. Among routine laboratory blood tests that we assessed as secondary outcome, low Hb, albumin, and TLC, and high creatinine, potassium, troponin T, and CRP have all been reported be associated with poor outcome in hip fracture patients [3,30,31,32].

The pattern of alterations in HMGB1 and IL-6 observed in our present study was similar to the observations of Giannoudis et al. [9], who reported an increase in HMGB1 later in the clinical course compared to that of IL-6. Although we were unable to measure cytokine levels beyond POD2, our results demonstrated that while IL-6 peaked on POD1 and started to decrease uniformly in all groups on POD2, HMGB-1 continued to increase in the propofol group while decreasing slightly in the desflurane and spinal groups on POD2. Unfortunately, we were unable to establish the predictive value or correlations of these cytokines with different modes of anesthesia. This also applied to the other laboratory blood tests that were evaluated in the present study. However, it is worth noting that there was a steady decrease in Hb in all groups up to POD2 and a trend for increases in troponin T and CRP after surgery.

Most existing studies have compared outcomes between regional and general anesthesia. However, the use of propofol-based TIVA has been reported to have positive outcomes compared to those following volatile anesthetics, especially for cognitive function in the elderly [33,34,35]. Propofol has antioxidant and anti-inflammatory properties [36], and can decrease the production of cytokines including IL-6 [37]. We, thus, further divided the method of general anesthesia into propofol-based TIVA and desflurane-based balanced anesthesia. However, we did not observe any difference in the incidence of postoperative delirium among groups in the present study, potentially because we only collected data on delirium and not postoperative cognitive dysfunction (POCD). Adding neuropsychological testing before and after surgery would have provided deeper insight into the ability of propofol anesthesia to improve cognitive function after hip fracture surgery in elderly patients. Moreover, there were no differences in perioperative HMGB1, IL-6, and other routine laboratory tests with propofol anesthesia compared to those of other anesthesia methods. One possible explanation for this may be that the dose of propofol used in the patients was relatively small due to short operation times and frailty of the elderly patients enrolled in this study. Moreover, the lack of any difference in outcome between three anesthetic techniques may have been different in other patient populations. The prospect of anesthesia being able to affect outcome after surgery is especially exciting in the field of oncology. Both propofol and regional anesthesia have been suggested to have immunomodulatory effects during the perioperative period in cancer patients [38,39], which ultimately may lead to improvement in recurrence and long-term survival [40].

The main differences that were observed among the three groups were significantly lesser amounts of intraoperative crystalloids administered and smaller number of patients that required vasopressors in the spinal group compared to that in both the desflurane and propofol groups. These results are similar to that of Biboulet et al. [41], where continuous spinal anesthesia was found to provide better hemodynamic stability than general anesthesia with propofol TCI or sevoflurane in elderly patients undergoing hip fracture surgery. Messina et al. also reported that spinal anesthesia provided a more stable hemodynamic profile than that of general anesthesia with sevoflurane in elderly hip fracture patients. [42] Although the precise degree and duration of perioperative hypotension that must be avoided is unclear, there is a consensus that longer and more severe intraoperative hypotension is associated with postoperative morbidity and mortality [43,44,45]. However, we did not analyze the incidence of hypotensive events; rather, we focused on the number of patients in each group who required multiple boluses of ephedrine or phenylephrine/norepinephrine administration for hemodynamic control during surgery. Frail, elderly patients often require continuous infusions of vasopressors rather than intermittent bolus administrations, which makes recording the exact number of episodes of hypotension difficult.

The mortality rate observed in the present study was lower than previously reported mortality rates at 3 months postoperatively. The overall survival rate up to 3 months after surgery for hip fractures has been reported to be between 5.3% to 10.5% [6,46,47] regardless of method of anesthesia, whereas the incidence observed in the present study was 1.7% to 3.3% in all groups. The reason for this lower mortality rate at 3 months is unclear. As a single-center study, our institution receives a high volume of elderly hip fracture patients and has a dedicated surgical and anesthesia team for this patient population. All patients enrolled in the present study were managed by the same team of anesthesiologists, and the level of experience and prompt response to intraoperative adverse events may have contributed to the outcome.

The present study has several limitations, some of which are common to trials of similar design. First, to truly randomize the method of anesthesia, we were unable to include patients taking certain oral anticoagulants that are contraindications for regional anesthesia. Second, our results included survival up to 90 days after surgery, with no difference observed in survival among the three groups. However, as our sample size calculation was not conducted for the assessment of secondary outcomes, such as postoperative morbidity and mortality, these results should be interpreted with caution. Third, a longer period of postoperative follow-up would have been beneficial for assessing the longer-term survival rate of patients enrolled in our study. Finally, the long-term functional impact, with regards to mobilization and neurological function, including POCD and intraoperative BIS levels, were not evaluated in our study. Regardless of the lower short-term mortality rate observed in our study, a longer follow-up period would shed more light on the effects of different anesthesia methods on patient function.

In conclusion, no differences in perioperative HMGB1 or IL-6 levels were observed between geriatric hip fracture patients who received either balanced inhalation anesthesia with desflurane, TIVA with propofol, or spinal anesthesia with local anesthetics. Clinical outcomes and other biological test results were also comparable between groups. The sole statistically significant finding was a difference in intraoperative fluid balance and the use of vasopressors between groups, but its clinical significance is uncertain.

## Figures and Tables

**Table 1:** Patient characteristics.

	Desflurane (*n* = 60)	Propofol (*n* = 58)	Spinal (*n* = 58)	*p*-Value
Age (years)	79.4 ± 7.7	80.5 ± 6.7	81.6 ± 6.7	0.234
Male	13 (21.7%)	16 (27.6%)	17 (29.3%)	0.611
Body mass index (kg/m^2^)	22.5 (20.0, 24.8)	22.3 (19.6, 24.8)	22.8 (20.4, 25.1)	0.787
Comorbidities				
Dementia	12 (20.0%)	7 (12.1%)	10 (17.2%)	0.500
Previous CVA	17 (28.3%)	14 (24.1%)	11 (19.0%)	0.490
CAD	15 (25.0%)	14 (24.1%)	12 (20.7%)	0.843
COPD	2 (3.3%)	1 (1.7%)	5 (8.6%)	0.175
DM	24 (40.0%)	25 (43.1%)	18 (31.0%)	0.380
Hypertension	44 (73.3%)	43 (74.1%)	45 (77.6%)	0.853
Renal dysfunction	8 (13.3%)	12 (20.7%)	13 (22.4%)	0.405
Baseline NT-proBNP	267 (147, 747)	404 (140, 1022)	250 (168, 631)	0.642
Type of fracture				0.170
Femoral neck	26 (43.3%)	26 (44.8%)	36 (62.1%)	
Intertrochanteric	27 (45.0%)	29 (50.0%)	21 (36.2%)	
Subtrochanteric	3 (5.0%)	1 (1.7%)	0	
Other	4 (6.7%)	2 (3.4%)	1 (1.7%)	
Time from injury to surgery (h)	78.5 (46.8, 150.5)	58.0 (39.3, 138.8)	76.0 (52.0, 140.8)	0.278
Time from admission to surgery (h)	46.5 (24.0, 71.0)	42.0 (22.8, 70.0)	47.5 (24.0, 72.0)	0.951

Values are mean ±SD or median (interquartile range, IQR); CVA, cerebrovascular accident; CAD, coronary artery disease; COPD, chronic obstructive pulmonary disease; DM, diabetes mellitus; NT-proBNP, N-terminal pro-brain natriuretic peptide.

**Table 2:** Operative characteristics.

	Desflurane (*n* = 60)	Propofol (*n* = 58)	Spinal (*n* = 58)	Overall *p*-Value
Type of surgery				0.235
Bipolar hemiarthroplasty	29 (48.3%)	25 (43.1%)	34 (58.6%)	
Internal fixation	31 (51.7%)	33 (56.9%)	24 (41.4%)	
Anesthesia time (minutes)	110 (100, 145)	115 (100, 144)	108 (96, 134)	0.405
Fluid balance				
Crystalloid (mL)	600 (438, 850)	600 (450, 850)	500 (400, 688) *^,**†**^	0.006
Colloid (mL)	0 (0, 0)	0 (0, 0)	0 (0, 100)	0.227
Urine output (mL)	100 (50, 200)	100 (51, 150)	100 (30, 150)	0.412
Blood loss (mL)	100 (50, 200)	100 (50, 200)	85 (50, 138)	0.344
Transfusion				
Amount of pRBC (U)	0 (0, 0)	0 (0, 1)	0 (0, 0)	0.047
Vasopressor use	37 (61.7%)	34 (58.6%)	16 (27.6%) *^,**†**^	0.000

Values are number (percent) or median (IQR); pRBC, packed red blood cells. * *p* < 0.05 compared with the desflurane group. ^**†**^ *p* < 0.05 compared with the Propofol group. Vasopressor use: multiple boluses of ephedrine (>8 mg) or phenylephrine/norepinephrine administration.

**Table 3:** Postoperative morbidity and mortality.

	Desflurane (*n* = 60)	Propofol (*n* = 58)	Spinal (*n* = 58)	*p* Value
Hospital days	6.5 (4.8, 9.0)	7.0 (5.0, 9.0)	7.0 (5.0, 11.0)	0.416
ICU days	0.0 (0.0, 0.0)	0.0 (0.0, 2.0)	0.0 (0.0, 0.0)	0.049
Ventilator days	0.0 (0.0, 0.0)	0.0 (0.0, 0.0)	0.0 (0.0, 0.0)	0.792
Postoperative days	3.0 (3.0, 5.0)	3.0 (3.0, 6.0)	4.0 (3.0, 6.0)	0.573
In-hospital complications				
Pulmonary complications	4 (6.7%)	5 (8.6%)	4 (6.9%)	0.907
Cardiac complications	3 (5.0%)	2 (3.4%)	2 (3.4%)	0.883
Delirium	9 (15.0%)	8 (13.8%)	8 (13.8%)	0.977
Postoperative mortality				
In-hospital mortality	1 (1.7%)	0	2 (3.4%)	0.357
30-day mortality	2 (3.3%)	1 (1.7%)	1 (1.7%)	0.794
90-day mortality	3 (5.0%)	2 (3.4%)	3 (5.2%)	0.886

Values are number (percent) or median (IQR). ICU, intensive care unit. Pulmonary complications: Pneumonia, respiratory failure. Cardiac complications: Myocardial infarct, heart failure, new-onset arrhythmia after surgery. Delirium: Aggravation or new-onset delirium after surgery.
